# The Oxidative Stress Markers in the Erythrocytes and Heart Muscle of Obese Rats: Relate to a High-Fat Diet but Not to DJOS Bariatric Surgery

**DOI:** 10.3390/antiox9020183

**Published:** 2020-02-22

**Authors:** Bronisława Skrzep-Poloczek, Jakub Poloczek, Elżbieta Chełmecka, Agnieszka Dulska, Ewa Romuk, Maciej Idzik, Wojciech Kazura, Katarzyna Nabrdalik, Janusz Gumprecht, Jerzy Jochem, Dominika Marta Stygar

**Affiliations:** 1 Department of Physiology, Faculty of Medical Sciences in Zabrze, Medical University of Silesia, 40-751 Katowice, Poland; 2 Department of Rehabilitation, 3rd Specialist Hospital in Rybnik, 44-200 Rybnik, Poland; 3 Department of Statistics, Department of Instrumental Analysis, Faculty of Pharmaceutical Sciences in Sosnowiec Medical University of Silesia, 40-751 Katowice, Poland; 4 Department of Biochemistry, Faculty of Medical Sciences in Zabrze, Medical University of Silesia, 40-751 Katowice, Poland; 5 Independent Public Health Care, Opole Cancer Center prof. Tadeusz Koszarowski, 45-061 Opole, Poland; 6 Department of Internal Medicine, Diabetology and Nephrology in Zabrze, Medical University of Silesia, 45-061 Katowice, Poland

**Keywords:** high-fat diet, HF diet, bariatric surgery, oxidative stress, DJOS, antioxidants

## Abstract

Obesity and high-fat diet (HF) are prevalent causes of oxidative stress (OS). Duodenal-jejunal omega switch (DJOS) is a bariatric procedure used for body mass reduction, extensively tested in animal models. We studied the long-term impact of bariatric surgery and an HF diet on the oxidative stress markers in erythrocytes and heart muscles of rats. We analyzed superoxide dismutase (SOD), catalase (CAT), glutathione transferase (GST), glutathione reductase (GR), glutathione peroxidase (GPx) activity and malondialdehyde (MDA) concentration in DJOS or SHAM (control) operated rats fed with different dietary protocols (control diet (CD) and high-fat diet (HF)), before and after the surgery (CD/CD, HF/HF, CD/HF, and HF/CD). We observed higher erythrocytes CAT, GST and GPx activity in DJOS-operated (vs. SHAM) rats fed with an HF/HF diet. For DJOS-operated rats, erythrocytes CAT and GPx activity and MDA concentration were significantly lower in CD/CD group. We observed increased heart muscle GR activity in SHAM-operated rats (vs. DJOS bariatric surgery) fed with an HF/HF diet. Change from HF to CD diet increased heart muscle GPx activity after DJOS bariatric surgery. Heart muscle SOD activity was lower in HF/HF and CD/CD groups after DJOS bariatric surgery (vs. SHAM). DJOS surgery significantly reduced heart muscle MDA concentration in HF/HF and HF/CD groups (vs. SHAM). We conclude that the selected dietary patterns had a stronger impact on oxidative stress markers in erythrocytes and heart muscle than DJOS bariatric surgery.

## 1. Introduction

Oxidative stress is a phenomenon reflecting a negative impact that the loss of balance between prooxidative and antioxidative factors has on cells. Studies confirm that oxidative stress takes part in the development of obesity-related complication [1,2,3]. Elevated levels of reactive oxygen species (ROS) and the lower activity of antioxidative enzymes are indicators of oxidative stress [4,5,6]. Both obesity affecting individuals worldwide and a diet rich in fat are linked to oxidative stress and cause many unfavorable health consequences [4,5,7,8].

Erythrocytes are one of many types of cells in which redox imbalance may occur. The fatty acids in the membranes, the increased oxygen level and the hemoglobin presence inside make erythrocytes natural targets for free radicals [9]. The oxidative processes in the erythrocytes entail membrane injury, modifications of the membrane’s structural and functional elements, and changes in the membrane architecture. All these processes cause an increase in erythrocytes’ mean osmotic fragility and inhibition of Mg^2+^ and Ca^2+^ ATPase activities [10]. The erythrocytes’ survival rate in plasma of circulating blood depends on the structural and functional integrality of their membranes, which determines the mechanical behavior of the cells. Erythrocytes express several physiological defense mechanisms against intracellular oxidative stress, including cellular, enzymatic antioxidant systems: superoxide dismutase (SOD), catalase (CAT) and glutathione S-transferase (GST) [9,10]. Detoxification of hydroperoxides (by glutathione peroxidase (GPx) and catalase) and superoxide free radicals (by SOD) serves as important antioxidative markers in the cell [9,10].

The cardiac function is also impaired by oxidative damage to cellular proteins and membranes, leading to dysfunction of cardiomyocytes and/or their death through apoptosis and necrosis [11]. The heart muscle, due to its structure and function, uses more than 90% of the energy produced during mitochondrial respiration. A high-fat (HF) diet leads to the increased rate of fatty acid oxidation to meet the growing demand for adenosine triphosphate (ATP) needed for heart muscle contractions. That is why the mitochondrial dysfunction impairs the contractile performance of the heart [12] and, thus, obesity increases the risk of cardiovascular diseases [13].

Bariatric surgery is a treatment method which effectively reduces body weight and mortality [14,15]. Duodenal-jejunal omega switch (DJOS) is a kind of bariatric protocol with proximal loop duodeno-enterostomy that bypasses the foregut (foregut theory), and thus the hindgut is directly stimulated (hindgut theory) [16,17]. The advantage of DJOS is a bypass-like procedure, where the pylorus of the patient is saved. This modification prevents patients from symptoms characteristic for postgastrectomy conditions, such as dumping, diarrhea and dyspepsia [18,19]. Duodenal-jejunal omega switch (DJOS) is a very promising bariatric protocol that ameliorates glucose tolerance but is also a rather novel bariatric procedure [20,21,22,23]. That is why the long-term effects of this type of surgery are still studied, mainly in the animal models [20,21,22,23,24].

So far, several studies explored the influence of the diet, operation for weight loss and oxidative stress in animal models [20,21,22,23]. In these studies, the HF diet increased fat tissue amount [24] and oxidative stress levels in skeletal muscles [25], and operation for weight loss proved to diminish the oxidative stress in rats [26]. As far as we are concerned, only one study explored all three abovementioned components together, and it was our previous work performed on rats: we proved the influence of bariatric procedures and diet on the antioxidative enzymes activities and malondialdehyde (MDA) concentration in the soleus muscle [27]. This paper is the continuation of the complex research on the oxidative stress markers in different organs. So far, no studies related to models of oxidative stress markers, high-fat diet and bariatric procedures in the heart tissue and erythrocytes have been performed. This research aimed to investigate the effect of DJOS metabolic surgery combined with an HF and control (CD) diet on antioxidant status of the selected tissues. Based on our previous results [20,27,28], we hypothesized that the HF diet would induce oxidative stress and it would overcome beneficial effects of DJOS bariatric surgery.

In our experimental design, we applied the results from the human studies, showing that some patients failed to reduce the daily caloric intake after metabolic protocol, by keeping half of the rats on an HF diet, post-surgery. We also assumed that, after surgery, patients might change the dietary preferences from a low-calorie diet to more energy-dense food and vice versa. The present research studied the effects of DJOS surgery with a high-calorie diet on antioxidant status of the heart muscle and erythrocytes in rats, which, to the best of our knowledge, has not been reported to date.

## 2. Materials and Methods

### 2.1. Animals and Diets

The methodology of the experiment was the same as previously described [27]. We used 56 male 7-week-old Sprague-Dawley rats (Charles River Breeding Laboratories, Wilmington, MA, USA) weighing 200 ± 7 g. Animals were kept in standard conditions: light–dark cycle 12:12, temperature 22 °C and humidity 40–60%. They also had constant access to water and rat food. Animals were becoming obese after two months’ feeding with a high-fat diet (HF) (EF RAT /E15744/Ssniff Spezialdiäten GmbH, Soest, Germany) composed of 27% carbohydrate, 59% fat and 14% protein. The control group of rats was fed with the control diet (CD) composed of 4.9% fat, 24% protein, 7% crude ashes and 4.7% crude fiber (Provimi Kliba AG, Kaiseraugst, Switzerland). Rats fed with HF and CD diet received, respectively, 5.04 and 3.59 kcal/g (23 and 15 kJ/g) of daily energy.

The experimental procedures were approved by the Local Ethical Committee for Animal Experimentation of the Medical University of Silesia (58/2014) and conducted according to Directive 2010/63/EU.

### 2.2. Experimental Design

**Figure 1:**
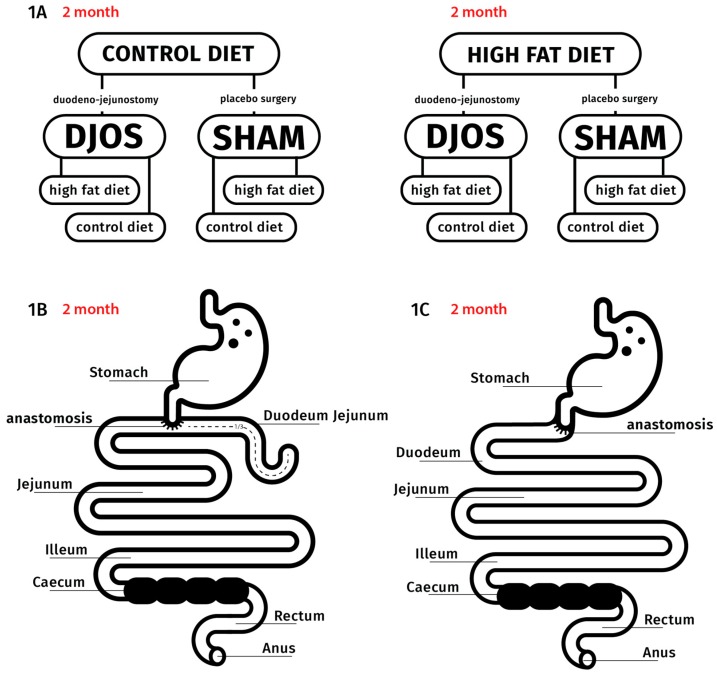
The experimental setup and surgeries performed: (**A**) design of the experiment, (**B**) DJOS (duodenal-jejunal omega switch) bariatric protocol and (**C**) SHAM (control) bariatric protocol.

After 7 days of acclimatization to the default conditions, the animals were randomly divided into two groups, CD (*n* = 28) and HF (*n* = 28), and kept on the respective diet for 2 months. After that time, the animals underwent one of the two types of the bariatric surgery: 14 animals underwent SHAM (control) procedure, and the other 14 underwent DJOS procedure (Figure 1A). After the procedure, half of the rats (*n* = 7) had the diet changed, and half of them remained on the same diet as previously (Figure 1A). Two months after DJOS and SHAM procedures, we collected the blood and heart tissue samples. The experimental procedures complied with the “3Rs” (Replacement, Reduction and Refinement) rule for performing more humane animal research [29].

### 2.3. Bariatric Procedures

The DJOS (duodenal-jejunal omega switch) procedure was done as previously reported [30]. Before DJOS surgery, the animals were anaesthetized with 2% isoflurane (AbbVie Deutschland GmbH & Co. KG, Wiesbaden, Germany). During the procedure: the animals maintained spontaneous breathing, and oxygen flow of 2 L/min was applied; we administered Xylazine (5 mg/kg, intraperitoneal (i.p.); Xylapan, Vetoquinol Biovet, Gorzów Wielkopolski, Poland) for analgesia and gentamicin (10 mg/kg, intramuscular (im); KRKA, Warszawa, Poland) for antibiotic prophylaxis. The entire duodenum and proximal jejunum were bypassed, whereas the stomach was left intact. The separation spot between the duodenum and the stomach was below the pylorus. The distal part of duodenum was closed with Prolene 6/0 (Ethicon, Somerville, New Jersey, United States). The duodeno-enterostomy was positioned above the Treitz ligament, located approximately at one-third of the total small-bowel length. The duodenojejunostomy was a hand-sewn (6-0 sutures), continuous end-to-side simple antecolic, extramucosal anastomosis. Carprofen (4 mg/kg, subcutaneous (sc); Rimadyl, Pfizer, Zürich, Switzerland) was used for analgesia for 3 consecutive days after the procedure.

To perform the SHAM (control) procedure, transections and re-anastomosis of the gastrointestinal tract were made at the analogous locations where enterotomies were performed for the DJOS so that the physiological passage of the food through the intestines could be maintained (Figure 1B,C). The anesthesia, analgesia, antibiotic prophylaxis and sutures applied were the same as for the DJOS procedure.

### 2.4. Blood Collection

Two months after the surgery, we collected blood samples (5 mL) from the right ventricle to the tubes with ethylenediaminetetraacetic acid (EDTA). Then we centrifuged the samples (5000 rpm, 10 min, 4 °C), to separate the erythrocytes, and washed the pellet with buffer solution (0.01 M PBS, 0.14 M NaCl, pH 7.4) three times. The separated erythrocytes were chilled to 4 °C and then stored at −80 °C, until the analysis. Before the analysis, the red blood cells were thawed, diluted with distilled water, and then they were chilled to 4 °C.

### 2.5. Tissue Collection

Two months post-surgery, we harvested 100 mg of cardiac tissue from the left ventricle and put it in 1 mL of a homogenizing buffer with protease inhibitors. The heart tissue was homogenized (1:10 *w*/*v*) in 0.9% NaCl with a glass homogenizer (Potter-Elvehjem PTFE, Sigma-Aldrich, Darmstadt, Germany) and then sonicated (Virsonic 100, VirTis, Gardiner, NY, USA). The lysate was centrifuged for 10 min, at 4000 rpm, at 4 °C, and treated as one independent sample. The tissue samples were then frozen and stored at −80 °C, until the analysis.

### 2.6. Oxidative Stress Markers’ Analysis

We analyzed the state of the antioxidant system in the heart tissue and erythrocytes. We determined the activity of total superoxide dismutase activity (SOD), catalase (CAT), glutathione reductase (glutathione disulfide reductase, GR, GSR), glutathione transferase (GST) and glutathione peroxidase (GPx). The lipid peroxidation was assessed by measuring malondialdehyde (MDA) concentration. In the heart muscle, we additionally determined the activity of Mn-dependent superoxide dismutase (MnSOD) and Cu–Zn superoxide dismutase activity (CuZnSOD).

#### 2.6.1. Glutathione Reductase (GR) Activity (EC 1.8.1.7)

GR activity was determined by using the kinetic method and is expressed as μmoles of Nicotinamide adenine dinucleotide phosphate (NADPH) utilized in 1 min 1 g of hemoglobin (IU/g Hb) for hemolysate or per 1 g of protein (IU/g protein) for heart muscle samples [31]. This method is based on changes in the concentration of NADPH that react with oxidized glutathione. The changes in absorbance at 340 nm were measured with a PERKIN ELMER Victor X3 reader (PerkinElmer, Inc., Waltham, Massachusetts, United States).

#### 2.6.2. Catalase (CAT) Activity (EC 1.11.1.6)

CAT activity was assessed, using the Aebi method [32]. In this method, the hemolysate or heart muscle homogenate is mixed with perhydrol in 50 mM TRIS/HCl buffer, pH 7.4, and the reaction is started by adding freshly prepared hydrogen peroxide. The rate of decomposition of hydrogen peroxide can be measured spectrophotometrically at 240 nm. CAT activity is expressed as units per 1 g of hemoglobin (IU/g Hb) for hemolysate, or per 1 g of protein (IU/g protein) for heart muscle samples.

#### 2.6.3. Glutathione Peroxidase (GPx) Activity (EC 1.11.1.9)

GPx activity was measured by using the kinetic method [33], with t-butyl peroxide as a substrate. In this reaction, oxidized glutathione (GSSG) is regenerated in the presence of glutathione reductase (GR) and NADPH. GPx activity was expressed as μmoles of NADPH oxidized in 1 min per 1 g of hemoglobin (IU/g Hb) for hemolysate or per 1 g of protein (IU/g protein) for heart muscle samples.

#### 2.6.4. Glutathione-S Transferase (GST) Activity (EC 2.5.1.18)

GST activity was estimated, using the Habig and Jakoby kinetic method [34]. The reaction mixture containing reduced glutathione was added to hemolysate/heart muscle samples. After initial stabilization, 1-chloro-2,3-dinitrobenzene (in ethyl alcohol solution) was added, and absorbance changes were monitored, using a PERKIN ELMER Victor X3 reader, at 340 nm wavelength, for at least 3 min. GST activity was expressed as μmoles of thioether formed within 1 min per 1 g of hemoglobin (IU/g Hb) for hemolysate, or per 1 g of protein (IU/g protein) for heart muscle samples.

#### 2.6.5. Superoxide Dismutase (SOD) Activity (EC 1.15.1.1)

Total SOD activity was measured, using the Oyanagui method [35]. In this method, xanthine oxidase catalyzes the production of superoxide anion that reacts with hydroxylamine to produce nitroso ion. The latter combined with n-(1-naphthyl)ethylenediamine and sulfanilic acid gives a color combination that can be measured spectrophotometrically.

Potassium cyanide (KCN) inhibits CuZnSOD activity; hence, CuZnSOD activity was assessed by calculating the difference between total SOD and MnSOD activity. Total SOD activity was presented as nitrite units (NU) per mg of hemoglobin/protein. One NU is 50% blockage of nitrite ions formation as described by Oyanagui [35].

#### 2.6.6. Malondialdehyde (MDA) Concentration

MDA concentration was measured by using the spectrophotometric method (wavelengths: 552 nm for emission and 515 nm for excitation; Perkin Elmer LS45 spectrofluorimeter, (PerkinElmer, Inc., Waltham, Massachusetts, United States) by Ohkawa et al. [36] and standard curve prepared for 1,1,3,3-tetraethoxypropane—the product of malondialdehyde and thiobarbituric acid reaction. MDA concentration was expressed in μmol/g protein for heart muscle and μmol/g Hb for erythrocytes.

#### 2.6.7. Protein Concentration

Hemoglobin concentration in hemolysates was estimated according to a modified Drabkin method [37]. Protein concentration in the heart muscle tissue was assessed according to the Lowry method [38].

### 2.7. Statistical Analysis

Statistical analysis was performed, using STATISTICA 12.5 PL (StatSoft, Cracow, Poland). The mean value ± SD (for a normal distribution) was used, and median with lower–upper quartile range (for data with skewed or non-normal distribution) was chosen to express interval data. The Shapiro–Wilk test evaluated the distribution of variables and the quantile–quantile plot, and homogeneity of variances was checked by using the Levene’s test. The U Mann–Whitney test, the non-parametric Kruskal–Wallis test, or the two-way parametric ANOVA with post hoc contrast analysis was used for comparison of data. Logarithmic transformation for skewed data distribution was performed before analysis. A *p* < 0.05 was considered statistically significant, and all of the tests were two-tailed.

## 3. Results

**Figure 2:**
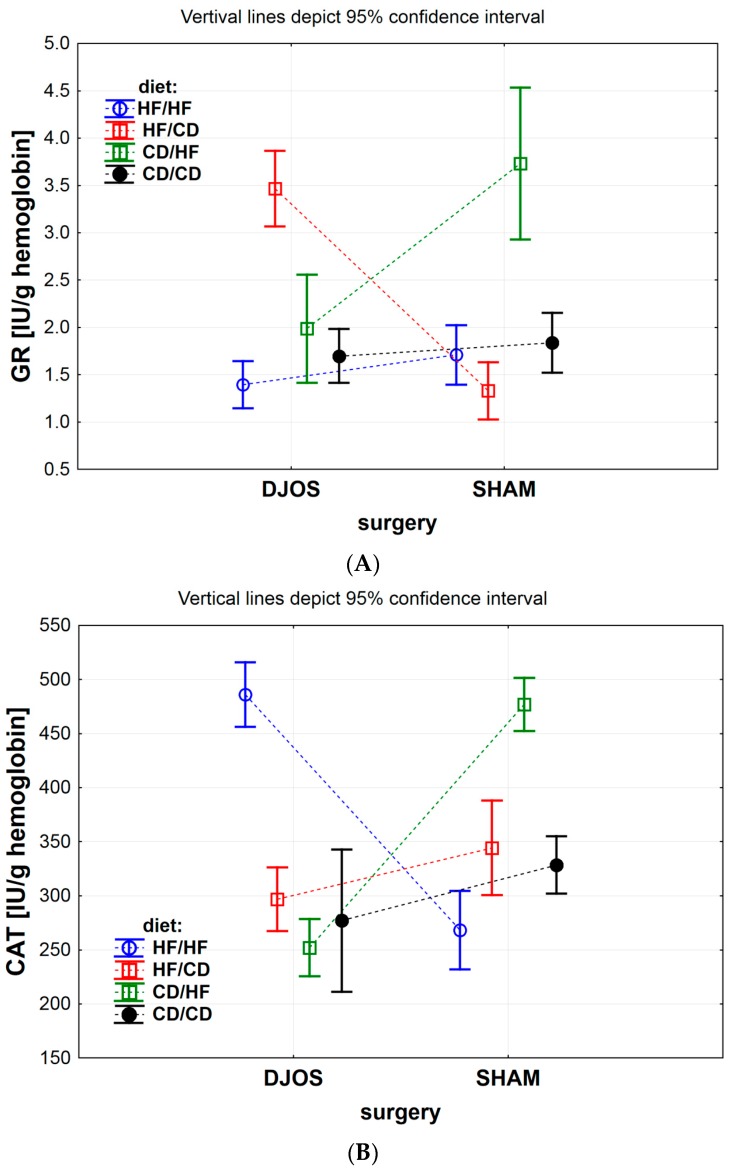

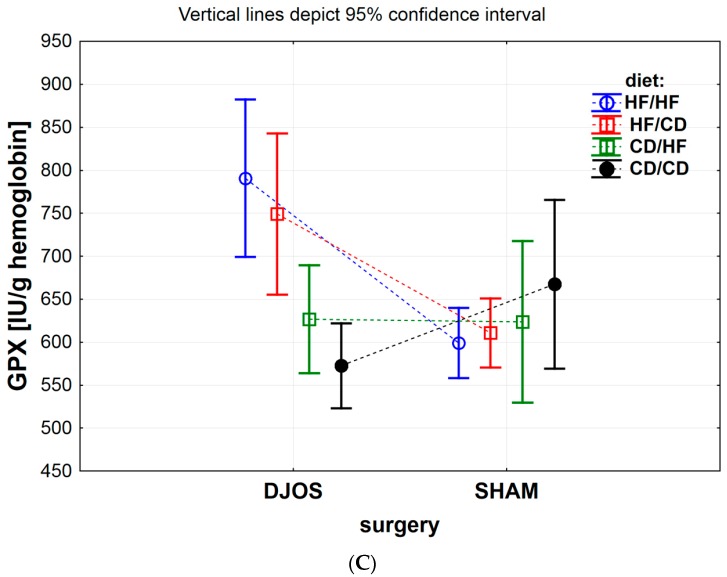
(**A**) Mean glutathione reductase (GR) activity (IU/g) in erythrocytes of rats from groups fed with high-fat (HF) and/or to (CD) control diet, before and after DJOS (duodenal-jejunal omega switch) or SHAM (control) surgery. (**B**) Mean catalase (CAT) activity (IU/g) in erythrocytes of rats from groups fed with high-fat (HF) and/or to (CD) control diet, before and after DJOS (duodenal-jejunal omega switch) or SHAM (control) surgery. (**C**) Mean glutathione peroxidase (GPx) activity (IU/g) in erythrocytes of rats from groups fed with high-fat (HF) and/or to (CD) control diet, before and after DJOS (duodenal-jejunal omega switch) or SHAM (control) surgery.

**Figure 3:**
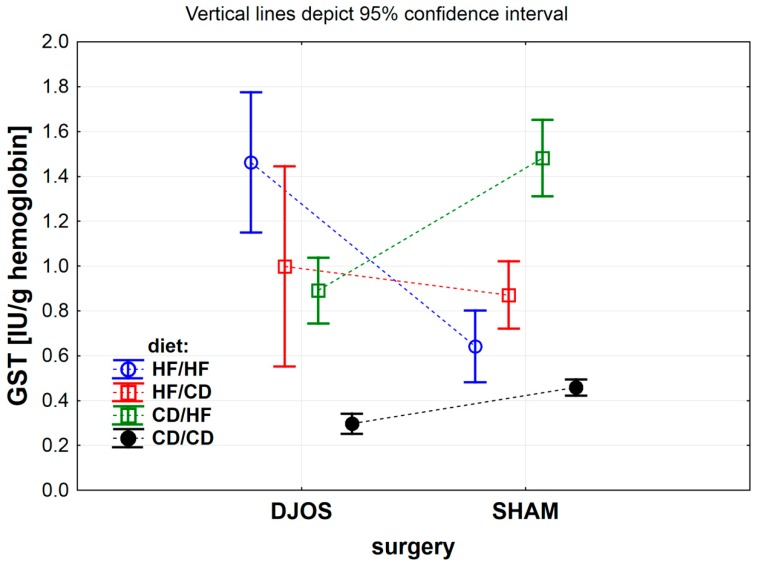
Mean glutathione S-transferase (GST) activity (IU/g) in erythrocytes of rats from groups fed with high-fat (HF) and/or to (CD) control diet before and after DJOS (duodenal-jejunal omega switch) or SHAM (control) surgery.

**Figure 4:**
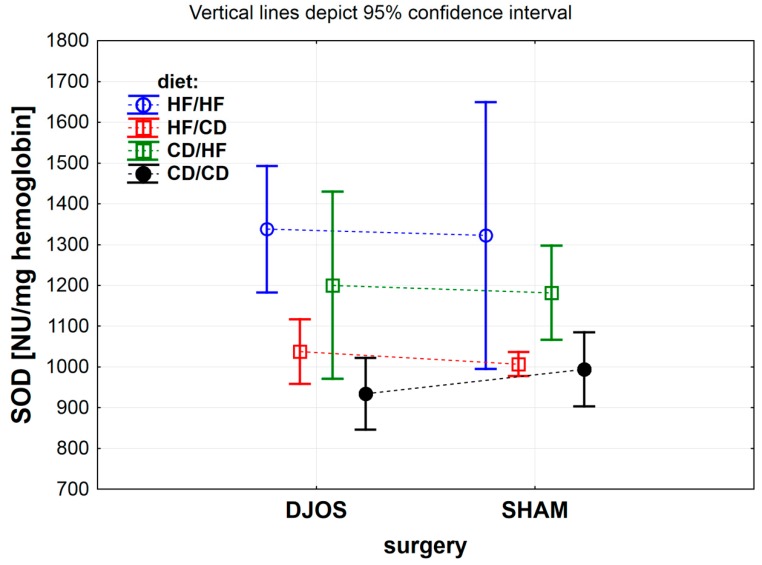
Mean total superoxide dismutase (SOD) activity (NU/mg) in erythrocytes of rats from groups fed with high-fat (HF) and/or to (CD) control diet, before and after DJOS (duodenal-jejunal omega switch) or SHAM (control) surgery.

**Figure 5:**
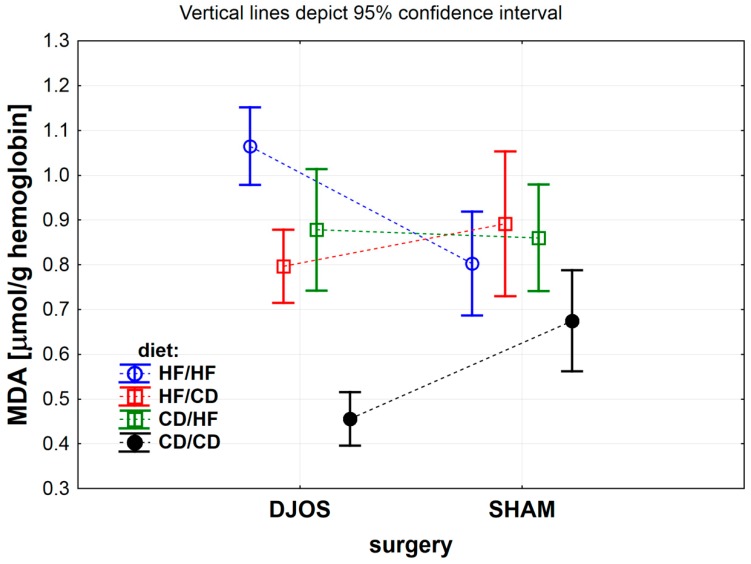
Mean malondialdehyde (MDA) concentration (μmol/g) in erythrocytes of rats from groups fed with high-fat (HF) and/or to (CD) control diet, before and after DJOS (duodenal-jejunal omega switch) or SHAM (control) surgery.

The effect of DJOS and SHAM bariatric procedures on antioxidative systems of the erythrocytes is presented in Figure 2, Figure 3, Figure 4 and Figure 5, and the results for heart tissue are presented in Table 1. The effect of diet used pre- and post-surgery, in relation to DJOS and SHAM procedures, on antioxidative systems of the erythrocytes and the heart muscle is presented in Table 2.

### 3.1. Erythrocytes

#### 3.1.1. Erythrocytes Glutathione Reductase (GR) Activity

GR activity in the erythrocytes of all experimental groups of animals that underwent DJOS and SHAM procedure was related to the diet used in the experiment (Figure 2A DJOS vs. SHAM, *p* < 0.001) and interaction between surgery and diet (Figure 2A DJOS vs. SHAM, *p* < 0.001), but it did not relate to the surgery itself (Figure 2A DJOS vs. SHAM, *p* = 0.897). When comparing the impact of the diet on GR activity between DJOS-operated and SHAM-operated animals, we noticed that the difference was significant for the HF/CD and CD/HF groups (Figure 2A and Table 2). GR activity of the DJOS-operated HF/CD group was significantly higher than in SHAM-operated HF/CD diet group, whereas GR erythrocyte’s activity in the DJOS-operated CD/HF group was significantly lower than in SHAM-operated CD/HF animals. The GR activity in erythrocytes of animals from the HF/HF and CD/CD study groups was at the same level in DJOS- and SHAM-operated rats.

GR erythrocyte’s activity significantly differed among DJOS-operated rats, with the highest activity in the HF/CD group and the lowest in the HF/HF group (Figure 2A and Table 2).

In SHAM-operated rats, the highest GR erythrocyte’s activity was observed in CD/HF group, while the lowest was in HF/CD group (Figure 2A and Table 2).

#### 3.1.2. Erythrocytes Catalase (CAT) Activity

The type of diet applied in all studied groups (Figure 2B DJOS vs. SHAM, *p* < 0.001), the type of surgery (Figure 2B DJOS vs. SHAM, *p* < 0.05) and their mutual relation (Figure 2B DJOS vs. SHAM, *p* < 0.001) significantly influenced CAT activity.

Erythrocytes CAT activity in the DJOS-operated rats significantly differed when compared to CAT activity in SHAM-operated animals (Figure 2B and Table 2). It was significantly higher in the HF/HF group and significantly lower in the HF/CD group, CD/HF and CD/CD of DJOS-operated animals, when compared to the respective diet groups of animals that underwent SHAM surgery (Figure 2B and Table 2).

In the DJOS-operated rats, the erythrocytes CAT activity differed significantly among studied dietary groups, i.e., HF/HF and CD/HF, HF/HF and HF/CD, and HF/HF and CD/CD, with the lowest activity measured for the CD/HF diet group and the highest measured for the HF/HF group (Figure 2B and Table 2).

Erythrocytes CAT activity also differed significantly among studied diet groups of the animals that underwent SHAM surgery (Figure 2B and Table 2), but in this group of animals, the trend was the opposite: the lowest value of CAT activity was measured for the HF/HF group and the highest for the CD/HF group (Figure 2B and Table 2).

#### 3.1.3. Erythrocytes Glutathione Peroxidase (GPx) Activity

The dietary pattern (Figure 2C DJOS vs. SHAM, *p* < 0.05), metabolic procedure (Figure 2C DJOS vs. SHAM, *p* < 0.01) and interaction between these two factors used in all experimental groups (Figure 2C DJOS vs. SHAM, *p* < 0.001) had a significant impact on the GPx activity in the erythrocytes. When comparing the animals that underwent DJOS surgery with these after SHAM procedure, we noticed significantly higher GPx activity in the HF/HF and HF/CD groups, and significantly lower activity in the CD/CD group for the DJOS-operated rats (Figure 2C and Table 2).

Erythrocytes GPx activity measured for DJOS-operated rats from four dietary groups differed significantly for the HF/HF and CD/HF, HF/HF and CD/HF, HF/CD and CD/HF, and also for the HF/CD and CD/HF group. The lowest GPx activity was noted for the CD/CD group, while the highest for the HF/HF diet group (Figure 2C and Table 2).

Erythrocytes GPx activity in SHAM-operated rats did not differ significantly for all four dietary groups (Figure 2C and Table 2).

#### 3.1.4. Erythrocytes Glutathione-S-Transferase (GST) Activity

The inter-group comparison of erythrocytes GST activity did not depend on the type of the surgery performed (Figure 3 DJOS vs. SHAM, *p* = 0.417) but depended on the diet the rats were fed (Figure 3 DJOS vs. SHAM, *p* < 0.001) and the interaction between the diet and the type of surgery performed (Figure 3 DJOS vs. SHAM, *p* < 0.001). GST activity in the group of animals that underwent the DJOS procedure was significantly higher in the HF/HF group and lower in the CD/HF groups, when compared with the SHAM-operated rats (Figure 3 and Table 2).

Erythrocytes GST activity of animals that underwent DJOS surgery differed significantly in the HF/CD and CD/CD, HF/HF and HF/CD, CD/HF and CD/CD, and also in the HF/HF and CD/HF dietary groups. The highest GST activity was observed in the HF/HF dietary group, and the lowest GST activity was measured in erythrocytes of animals fed with the CD diet pre- and post-surgery (Figure 3 and Table 2).

Erythrocytes GST activity in SHAM-operated rats significantly differed for the HF/CD and CD/HF, HF/HF and CD/HF, HF/CD and CD/CD, and also for the CD/HF and CD/CD dietary groups (Figure 3 and Table 2).

#### 3.1.5. Erythrocytes Total Superoxide Dismutase (SOD) Activity

The total SOD activity, compared between study groups of DJOS- and SHAM-operated animals, was influenced only by the diet type used pre- and post-surgery (Figure 4 DJOS vs. SHAM, *p* < 0.001). The type of the metabolic procedure and interaction between studied factors had no impact on the total SOD activity measured in the rats’ erythrocytes (Figure 4 DJOS vs. SHAM, *p* = 0.982 and *p* = 0.912, respectively).

Total SOD activity measured in the erythrocytes of DJOS-operated rats differed significantly for the HF/HF and CD/HF, HF/HF and HF/CD, and also for the HF/HF and CD/CD dietary groups. The lowest value of total SOD activity was observed for the CD/CD dietary group, whereas the highest value was measured for the HF/HF group (Figure 4 and Table 2).

Among SHAM-operated rats, the erythrocytes total SOD activity differed between the HF/HF and CD/CD, and the HF/HF and HF/CD groups. The highest SOD activity was noted for the HF/HF dietary group, whereas the lowest was noted for the CD/CD dietary group (Figure 4 and Table 2).

#### 3.1.6. Erythrocytes Malondialdehyde (MDA) Concentration

In DJOS vs. SHAM study groups, the lipids peroxidation level significantly depended on the diet used before and after the surgery (Figure 5 DJOS vs. SHAM, *p* < 0.001), as well as on the interaction between surgery and diet (Figure 5 DJOS vs. SHAM, *p* < 0.001), but not on the type of the bariatric procedure per se (Figure 5 DJOS vs. SHAM, *p* = 0.788). Erythrocytes MDA concentration significantly increased in DJOS-operated rats from the HF/HF dietary group, when compared with the rats from the same dietary group of rats that underwent SHAM procedure. The opposite change was noted for the CD/CD study group (Figure 5 and Table 2).

Among DJOS-operated rats, the highest erythrocytes MDA concentration was detected in the HF/HF dietary group, whereas the lowest for the CD/CD dietary group (Figure 5 and Table 2).

In SHAM-operated rats, the erythrocytes MDA concentration differed significantly between the HF/CD and CD/CD, HF/HF and CD/CD, and also between the CD/HF vs. CD/CD dietary groups. The highest MDA concentration was measured for the HF/CD group, whereas the lowest MDA concentration for the CD/CD group (Figure 5 and Table 2).

### 3.2. Heart Muscle

#### 3.2.1. Heart Muscle Glutathione Reductase (GR) Activity

Heart muscle GR activity was significantly influenced both by the diet and the surgery type and additionally by their mutual interaction (Table 1 DJOS vs. SHAM; Table 2).

The highest values of GR activity in the heart muscle were measured in the groups with an HF diet used pre- and post-DJOS and SHAM procedures, while the lowest activity of this enzyme was measured for groups of rats fed pre- and post-operatively with a CD diet. We observed that significantly higher levels of GR activity in the heart muscle were measured for SHAM-operated animals, and lower levels of GR activity were measured for DJOS-operated animals in the groups where an HF diet was used (HF/HF, HF/CD and CD/HF) (Table 1 and Table 2).

The highest heart muscle GR activity was observed in the HF/HF group of DJOS-operated rats, when compared with other dietary groups (Table 1 and Table 2). The same result was observed for rats from SHAM-operated groups.

#### 3.2.2. Heart Muscle Catalase (CAT) Activity

Heart muscle CAT activity was significantly influenced by the dietary pattern diet used in the experiment, by the type of surgery performed and by the interaction between these two factors (Table 1 DJOS vs. SHAM; Table 2).

The effect of the surgery was most clearly observed in the HF/HF group, where the heart muscle CAT activity was fivefold higher in the SHAM-operated rats, when compared with DJOS-operated animals (Table 1 and Table 2).

The HF/HF dietary plan significantly reduced heart muscle CAT activity in comparison to the other dietary groups of DJOS-operated rats. Changing the diet from HF to CD after DJOS procedure significantly altered heart muscle CAT activity: It was almost two times higher in comparison with the HF/HF dietary group. The effect of surgery on heart muscle CAT activity was visible in all DJOS-operated animals, except for the HF/HF group, where a strong interference of the diet was observed.

Th HF diet used pre- and post-SHAM surgery significantly changed heart muscle CAT activity: It was three times higher than in the HF/CD, CD/HF and CD/CD dietary groups (Table 1 and Table 2).

#### 3.2.3. Heart Muscle Glutathione Peroxidase (GPx) Activity

Inter-group analysis showed that heart muscle GPx activity depended on the surgery and the dietary pattern (Table 1 DJOS vs. SHAM; Table 2).

The general pattern of heart muscle GPx activity was similar for both types of procedure. Heart muscle GPx activity was significantly elevated in the HF/CD dietary group of DJOS-operated animals when compared with to the same dietary group of SHAM-operated animals (Table 1 and Table 2).

Among the DJOS-operated animals, heart muscle GPx activity of the HF/CD group was the highest, while GPx activity of the HF/HF group was the lowest. After the DJOS procedure, a switch of the dietary pattern from HF to CD significantly increased GPx activity, while the HF/HF dietary pattern reduced it significantly, when compared to the CD/CD group (Table 1 and Table 2).

We observed no significant differences between the different dietary protocols of SHAM-operated animals.

#### 3.2.4. Heart Muscle Glutathione-S-Transferase (GST) Activity

Heart muscle GST activity was similar in all dietary groups both after DJOS and after SHAM surgery. Hence, no significant changes in heart muscle GST activity were observed, so the multiple comparisons in contrast analysis were not performed (Table 1 DJOS vs. SHAM; Table 2).

#### 3.2.5. Heart Muscle Total Superoxide Dismutase (SOD) Activity

Heart muscle total SOD activity was significantly influenced by the dietary protocol, by the type of surgery performed and by the interaction between these two parameters (Table 1 DJOS vs. SHAM; Table 2).

Heart muscle total SOD activity was significantly lower in the HF/HF, CD/HF and CD/CD diet groups of DJOS-operated rats than in the respective groups of SHAM-operated rats (Table 1 and Table 2). DJOS surgery significantly reduced total SOD activity in the HF/HF and CD/CD dietary groups, when compared with the same group in SHAM-operated rats.

The diet change from CD to HF significantly increased heart muscle total SOD activity measured for DJOS-operated animals (Table 1 and Table 2). Each diet change, from HF to CD and from CD to HF, significantly increased heart muscle total SOD activity, when compared to the groups with no change in the diet in DJOS-operated rats (Table 1 and Table 2).

In SHAM-operated animals, an HF diet used pre- and post-surgery strongly increased total SOD activity when compared to the groups with the changed dietary pattern. In contrast to the DJOS-operated animals, change in the dietary pattern per se strongly decreased total SOD activity measured in the heart muscle of SHAM-operated animals (Table 1 and Table 2).

#### 3.2.6. Heart muscle Mn-Dependent Superoxide Dismutase (MnSOD) Activity

Both DJOS and SHAM surgery and the dietary pattern had a significant impact on heart muscle MnSOD activity; however, the interaction between these two factors did not influence on the enzyme activity (Table 1 DJOS vs. SHAM; Table 2).

Significantly higher levels of heart muscle MnSOD activity were observed in the DJOS-operated rats fed with the HF/HF diet, in comparison to SHAM-operated animals.

The change from HF to CD diet resulted in increased heart muscle MnSOD activity, when compared with rats fed with HF pre-and post-DJOS procedure (Table 1 and Table 2).

In SHAM-operated animals, the heart muscle MnSOD in the HF/HF group was significantly reduced, when compared to other analyzed dietary groups (Table 1 and Table 2).

#### 3.2.7. Heart Muscle Copper–Zinc Superoxide Dismutase (CuZnSOD) Activity

A combination of the dietary pattern used pre- and post-bariatric protocol had a significant influence on CuZnSOD activity in the heart muscle of DJOS and SHAM-operated rats (Table 1 DJOS vs. SHAM; Table 2). Nevertheless, the activity of CuZnSOD did not depend on the type of surgery applied in the experiment (Table 1 and Table 2).

Heart muscle CuZnSOD activity in the groups with the changed dietary pattern (HF/CD and CD/HF) was significantly higher in DJOS-operated animals, when compared to SHAM-operated animals, whereas the enzyme activity in groups with no change in the diet (CD/CD and HF/HF) was higher in SHAM-operated animals when compared to those DJOS-operated (Table 1 and Table 2).

Heart muscle CuZnSOD activity of DJOS-operated rats was higher in the groups with a change in the dietary pattern (from HF to CD and from CD to HF), when compared to the groups with no change in the diet (HF/HF and CD/CD).

The activity of heart muscle CuZnSOD was significantly lower in the groups of animals fed with CD diet before and/or after SHAM procedure (CD/HF and CD/CD) when compared to animals kept on the HF diet pre- and post-operatively. Heart muscle CuZnSOD activity in the HF/CD group was significantly higher in comparison with the CD/HF group, and it was significantly lower than in CD/CD group of SHAM-operated rats (Table 1 and Table 2).

#### 3.2.8. Heart Muscle Malondialdehyde (MDA) Concentration

The process of lipid peroxidation in the heart muscle was significantly modified by the type of dietary pattern, by the bariatric protocol applied in the experiment and by the interaction between the diet and the surgery (Table 1 DJOS vs. SHAM; Table 2).

DJOS surgery significantly reduced MDA concentration in the heart muscle of animals fed with the HF/HF and HF/CD diet when compared with SHAM-operated animals (Table 1 and Table 2). MDA concentration in the heart muscle of animals from the CD/HF DJOS-operated group was significantly elevated when compared to the respective dietary group of SHAM-operated animals.

A change of diet from CD to HF resulted in a significant increase of MDA concentration in the heart muscle of DJOS-operated rats, when compared to other dietary groups of animals that underwent the same procedure (Table 1 and Table 2).

An HF diet used before and after SHAM surgery (HF/HF) and change from HF to CD diet (HF/CD) resulted in significantly higher MDA concentration when compared to the groups of rats fed with the CD diet before the surgery (Table 1 and Table 2). The HF/CD dietary pattern resulted in the highest MDA concentration within the SHAM-operated groups of rats.

## 4. Discussion

This paper is a continuation of the recently presented project by Skrzep-Poloczek et al. [27] that aimed to assess the relationship between bariatric surgery, high-fat (HF) diet and oxidative stress. Since the field of bariatric surgery develops intensively, systematic research is compulsory. Here we report the impact of bariatric surgery and high-fat diet on the oxidative stress markers in blood and heart muscle, in the experimental animal model [27,30].

### 4.1. Erythrocytes

We found that the influence of the diet on the erythrocytes glutathione reductase (GR) activity in DJOS (duodenal-jejunal omega switch) and SHAM-operated (control) rats was significantly higher in groups with the change in diet after the surgery (CD/HF and HF/CD, where CD stands for control diet) than in the rats fed with the same diet before and after surgical procedure (CD/CD and HF/HF). Endogenous defense mechanisms are often not effective enough to completely counteract or neutralize free radicals triggered by a high-fat diet [39]. This work may confirm the hypothesis that changes in GR activity profile may be assessed in relation to diverse dietary patterns, but not necessarily to DJOS or SHAM bariatric surgery methods. The highest catalase (CAT) activity in the heart muscle of rats maintained on the HF diet before and after DJOS surgery may suggest that the deleterious effect of the HF diet is stronger than the protective effect of DJOS surgery. A similar situation was detected for SHAM-operated rats from the CD/HF group: the change to an HF diet after the surgery caused a significantly higher ROS production, which was not observed in other study groups. The highest susceptibility to lipid peroxidation was found in the erythrocytes of rats fed with the HF diet. Changes in erythrocytes GR activity, compared to other analyzed enzymes, might be considered a compensatory mechanism neutralizing the negative impact of reactive oxygen species (ROS) on erythrocyte’s metabolism and being triggered under the conditions of ketogenic and obesogenic diet. Glutathione peroxidase (GPx) activity is frequently analyzed in erythrocytes as a marker of hemoglobin autoxidation and also of a regular generation of ROS and hydrogen peroxide [40]. In human studies, age, gender, lifestyle and ageing determined the antioxidant enzymes activity [41]. In accordance with this research, the HF diet significantly surged GPx activity in animals that underwent DJOS surgery. Other studies showed that GPx activity positively correlated with the circulating levels of oxidized LDL [42]. We might assume that the observed boost in GPx erythrocyte’s activity neutralizes the potential damage caused by oxidant factors. It is also known that the increased amount of visceral adipose tissue leads to more intensive ROS production [43,44,45]. Moreover, elevated levels of fatty acids in plasma of obese adults appear to trigger enzymes activity [46]. A hypercholesterolemic, atherogenic diet affects cholesterol/phospholipid ratio of erythrocyte’s membrane, consequentially changing its structural integrity and thus increasing cells’ osmotic frailty [47]. In this work, erythrocytes glutathione S-transferase (GST) activity was strongly stimulated by the HF diet both in DJOS- and SHAM-operated rats. The increase in GST activity surged by nutritional exposure to high-fat content provides plausible mechanistic evidence that the diet and ROS-induced inflammation significantly influence different pathological processes.

Oxidative stress is an essential factor that modulates the progression of complications triggered by diet-induced obesity, such as impaired glucose tolerance, in rats. Rats maintained on an HF diet showed increased body mass and increased levels of oxidative stress markers, including total superoxide dismutase (SOD) activity [48,49]. Cellular enzymatic antioxidants play a supportive role in defense against oxygen free radicals, and superoxide dismutase is one of the essential anti-ROS enzymes. Hyperlipidemia is regarded as a serious clinical manifestation of HF diet fed to rats [50]. Our results show that erythrocytes’ total SOD activity depended on the HF diet but not on the surgery performed in the experiment.

Obesity is an unconstrained risk factor for intensive oxidative lipid destruction and reduced activity of cellular protective enzymes. No study, neither in animals nor in humans, has yet to analyze malondialdehyde concentration in erythrocytes after the bariatric procedure in relation to HF and CD diets. The results of this study prove that an HF diet enhances lipid peroxidation in erythrocytes, expressed by elevated levels of malondialdehyde (MDA) in hemolysate, independently from the surgery, and this harmful dietary impact is stronger than the protective effect of bariatric surgery. Taking into account the information presented above, we may hypothesize that erythrocyte’s antioxidative enzymes activity depends more on the type of diet used pre- and post-operatively and the enzyme investigated than on the bariatric surgery. The results imply that the HF diet is the least favorable method of therapy, while the control diet (CD) is more beneficial in respect of oxidant–antioxidant status following bariatric surgery.

In our previous research, we demonstrated that the dietary pattern selected pre- and post-DJOS and SHAM protocols determines levels of oxidative stress, the activity of enzymatic and non-enzymatic systems in the soleus muscle of rats [27]. The present study, which is a continuation of the previous experiment, confirms that the antioxidant status is associated with the type of diet used pre- and post-operatively. However, we have found that the oxidative stress enzymes responses to diet and operation stimuli in erythrocytes are different from those observed previously in the skeletal muscle. In the former experiment, we found that feeding rats with the CD or HF diet before and after the operation was related to the lowest level of antioxidative enzymes activity, while diet change after the operation, from CD to HF or the reverse, remarkably exacerbated the ROS production and lipid peroxidation [27]. The main observation coming up from the present study is that the HF diet used before and after DJOS operation, as compared to the SHAM procedure, led to a significant increase in some antioxidative enzymes activity (CAT, GPx and GST) and MDA concentration, while using the CD diet before and after the surgery resulted in significantly lower antioxidative enzymes activity (CAT and GPx) and MDA concentration. After DJOS surgery, the activity of all investigated enzymes (except for GR) and MDA concentration were increased in rats fed according to the HF/HF dietary pattern, in comparison with rats fed according to the CD/CD dietary pattern. Remaining on the HF diet after the surgery led to more disturbances in oxidative stress, as measured by activity of enzymes and MDA concentration in erythrocytes than remaining on the CD diet, while, in our previous experiment, the most detrimental to the soleus muscle of the studied rats changing was the diet from HF to CD or the reverse.

The results of the presented study indicate that, in regard to oxidative stress in erythrocytes, it does matter what kind of diet is used before and after the procedure, and the control diet is always the choice of preference. This has the practical implication: In the future, whenever bariatric surgery procedures are concerned, the type of diet should be analyzed, especially in relation to human subjects.

### 4.2. Heart Muscle

A mechanism underlying heart failure in obese patients is related to the altered fat tissue amount, inflammatory processes and changed cardiac physiology that is convoluted by co-morbidities [51]. Morbid obesity may increase metabolic demands and shift the cardiomyocyte substrate usage from fatty acids to glucose [51]. As a consequence, this can impair glucose uptake and utilization and fatty acid ß-oxidation, and it can also lead to mitochondrial dysfunction and reactive oxygen species (ROS) generation [52]. Increased glutathione reductase (GR) activity seems to be a defensive mechanism preventing the over-quenching of intracellular ROS required for insulin signaling [53]. The increased glutathione production protects cardiac muscle against electromechanical dysfunction and cell death and is strongly connected with higher GR activity [54]. Our results show that heart muscle GR activity was strongly influenced by both studied factors: the type of diet and surgery applied. We observed a similar pattern of GR activity in all analyzed combinations of diets for DJOS- and SHAM-operated rats; nevertheless, heart muscle GR activity in DJOS-operated groups was lower than in SHAM-operated ones. The highest activity of this enzyme was measured for the HF/HF groups. DJOS surgery significantly affects GR activity in the heart muscle, when compared to SHAM-operated animals. Knowing that reactive oxygen species are responsible for the changes in GR activity, we hypothesized that DJOS surgery had a protective effect on ROS production and thus reduced the deleterious effect of selected combinations of dietary patterns.

The heart is particularly sensitive to oxidative stress, having lower levels of ROS scavengers such as copper–zinc superoxide dismutase (CuZnSOD), catalase (CAT) and glutathione reductase, (GPx) when compared to other organs, especially to the liver [55]. CAT is one of the enzymes neutralizing H_2_O_2_, and thus its activation in SHAM-operated rats fed with HF diet might be connected with the reduction of H_2_O_2_ pool in vivo. Cardiac oxidative stress and high CAT activity levels are correlated with boosted fat metabolism, high-fat diet and obesity [56]. We report a decrease in CAT activity in DJOS-operated rats fed with an HF diet pre- and post-surgery, when compared to the respective groups of SHAM-operated rats. Heart muscle CAT activity after DJOS surgery remained unchanged in the groups with different dietary patterns, except for the HF/CD group, where the change of the diet increased its activity. A significant increase in heart muscle CAT activity was reported for the HF/HF dietary group of SHAM-operated arts. Other study shows that the HF diet impaired CAT activity during ischemia/reperfusion injury and reduced the capacitance of the myocardium to scavenge reactive oxygen species produced during this injury. Nevertheless, the change from HF to CD significantly downregulated catalase protein expression back to the control-diet level [57]. That observation goes along with our results for DJOS-operated rats: Heart muscle CAT activity in the HF/CD group was similar to CD/CD dietary group of SHAM-operated rats (control). We suggest that exclusion of the first part of the digestive tract from contact with high-energy condensed food stimulates a reduction in CAT activity. This can support the thesis that feeding rats with a control diet before and/or after the surgery improved antioxidant reserve of their hearts. DJOS surgery significantly reduced the deleterious effect of HF/HF dietary pattern measured by CAT activity in the heart muscle tissue.

Our study showed limited differences in heart muscle glutathione peroxidase (GPx) activity between DJOS and SHAM-operated animals. Only the HF/CD group of DJOS-operated rats showed higher GPx activity when compared to the respective group of SHAM-operated animals. That may suggest that a change of diet from HF to CD stimulates a glutathione-dependent response in the cardiomyocytes of DJOS-operated rats.

Studies performed by Hill and Signal [58] on the rat experimental model confirmed that cardiac failure and myocardial infarction are related to the antioxidant capacity deficit and increased oxidative stress. Other studies showed that bariatric procedures efficiently amended cardiovascular risk and diminished overall mortality [15,59]. Nevertheless, the mechanisms that control these cardiovascular ameliorations stay unclear. The reduction of the body mass after surgery also stimulates normalization of blood pressure and improvements to left ventricular mass [60,61]. The correlations between serum oxidative stress markers and the results after effort go along with the hypothesis that redox markers correlate with cardiac contractility and cardiac output, as well as with impairment of peripheral blood flow, hypoxia and skeletal muscle function [62,63,64]. It is known that mitochondrial respiration in the cells and tissues increases after gastric bypass and sleeve gastrectomy, when compared with the preoperative status of the patients [65]. However, the impact of dietary modifications on cardiovascular outcomes before and after bariatric surgery is still not fully studied [66]. Total superoxidase dismutase (SOD) activity is a marker of primary defense acting against oxygen-derived free radicals and is significantly induced by exposure to oxidative stress. Total SOD activity also maintains the aerobic conditions of the cell [67]. Other studies on the HF diet reported elevated SOD activity in HF-diet-induced obese rats, which may be understood as a compensatory adaptation to oxidative stress in high-fat-diet-induced obesity [68]. In our study, different dietary patterns pre- and post-DJOS and SHAM surgery had a strong impact on total SOD activity measured in heart muscle. A change from an HF diet to a CD and from a CD to an HF caused an increase in total SOD activity in DJOS-operated rats in comparison to the groups that remained on the same type of the diet for the duration of the experiment. Conversely, a change of the diet after SHAM procedure led to a decrease in total SOD activity. Regarding the total SOD and CuZnSOD activities, our results show that, in relation to DJOS surgery, a change of the diet from HF to CD and/or from CD to HF caused an increase in activity of those two distinct enzymes in comparison to the groups that remained on the same type of the diet. The activity of CuZnSOD was strongly related to the type of dietary patterns used in the experiment. We observed similar patterns of heart muscle Mn-dependent superoxide dismutase (MnSOD) activity after DJOS and SHAM. MnSOD, which is active in the mitochondria, is responsible for more than 70% of the heart muscle total SOD activity and more than 90% of this enzyme activity in cardiomyocytes. The remaining activity of total SOD is covered by CuZnSOD, which is typically working in the cytosol [69]. A strong influence on MnSOD was reported in anthracycline-induced cardiomyopathy. Systemic anthracycline treatment significantly downregulated the expression of MnSOD mRNA, of the respective protein and reduced its enzymatic activity for approximately 21 days [70]. In our study, a change of the diet caused an increase in the heart muscle MnSOD activity in the DJOS- and SHAM-operated animals. Exclusion of 1/3 of the small intestine and change of diet stimulated the total SOD, MnSOD and CuZnSOD activities, which are the main scavengers of anion superoxide (O_2_^−^). The cardiac oxidative stress, as demonstrated by increased MnSOD and CuZnSOD activities, was higher in DJOS-operated rats when compared to SHAM-operated ones. More intense adaptive processes could explain the discrepancy between DJOS- and SHAM-operated animals after severe stress caused by duodenal-jejunal exclusion and metabolic intervention.

Lipid peroxidation is considered to be an element of obesity-induced pathology [71]. Elevated malondialdehyde (MDA) levels were observed in the plasma of patients with symptoms of congestive heart failure [72]. Our results show that obesity increased lipid peroxidation in cardiac muscle was higher in SHAM-operated animals fed with the HF diet, before and after the surgery, than in DJOS-operated groups. The heart muscle MDA cardiac concentration was significantly reduced in rats fed with a CD diet. DJOS surgery significantly reduced the risk factor for increasing lipid peroxidation. A significant increase in MDA concentration in the HF/CD group may suggest that change from a CD to an HF diet after DJOS surgery reduces antioxidant defense mechanisms. DJOS surgery had a beneficial effect on the MDA concentration in the groups fed only with the atherogenic, HF diet. A switch of the diet from HF to CD and/or from CD to HF significantly increased the heart muscle MDA concentration in groups after both types of surgery.

Several studies on heart failure have provided consequential reports that oxidative stress enhances under the conditions of heart failure and contributes to disease progression [73]. The pivotal role of oxidative stress is also well explained in diabetic cardiomyopathy, endemic cardiomyopathy (Keshan disease) and Chagas disease [74,75,76,77]. The cardiomyopathy includes, among others, asymptomatic diastolic dysfunction and end-stage left ventricular (LV) dilation with reduced systolic function. Moreover, studies show that, in obese subjects, the probability of heart failure increases by 30%–100% [51]. Metabolic surgery substantially changes heart muscle geometry, function and symptoms connected to obesity cardiomyopathy [51]. The detailed mechanisms of the cardiac failure amelioration after bariatric surgery remain unexplained, but they are likely related to the effect of significant fat tissue reduction on cardiac workload, on inflammatory processes and to positive weight-loss independent changes in the entero-cardiac axis [51]. Lifestyle and environmental and epigenetic interactions reflect on myocardial remodeling and heart failure, in which the oxidative stress plays an important role [73]. In this paper, based on the animal model, we report that type of diet per se, besides the bariatric surgery, is the main driver regulating oxidative stress in the heart muscle. The DJOS surgery, along with selected dietary plans, may be used to moderate the effects of oxidative stress in the cardiac tissue.

## 5. Conclusions

The selected dietary patterns had a stronger impact on oxidative stress markers in erythrocytes and heart muscle than the bariatric surgery. The diet significantly stimulated all analyzed enzymatic and non-enzymatic parameters in erythrocytes, while surgery had a significant impact only on catalase (CAT) and glutathione peroxidase (GPx) activity. Long-term application of the control diet and the change of the diets after the surgery led to significant improvement in the response to the oxidative stress after DJOS surgery. Although DJOS surgery has a positive impact on the regulation of metabolic processes and the reduction of oxidative stress, the HF diet is still the primary determinant. Further studies need to include the type of diet as a significant component influencing the effects of bariatric surgery on oxidative stress makers.

## Figures and Tables

**Table 1:** The results of glutathione reductase (GR), catalase (CAT), glutathione peroxidase (GPx), glutathione S-transferase (GST), total superoxide dismutase (SOD), Mn-dependent superoxide dismutase (MnSOD), copper–zinc superoxide dismutase (CuZnSOD) activity and malondialdehyde (MDA) concentration in heart muscle of rats from study groups subjected to high-fat (HF) and/or to (CD) control diets. Descriptive statistics and results of two-way analysis of variance for inter-group comparison between DJOS-operated (duodenal-jejunal omega switch) and SHAM-operated (control) study groups. Results are presented as mean ± SD or median (lower–upper quartile). Statistical significance was set at *p* < 0.05.

Oxidative Stress Marker	DJOS-Operated				SHAM-Operated				*p* ANOVA		
	HF/HF	HF/CD	CD/HF	CD/CD	HF/HF	HF/CD	CD/HF	CD/CD	Group	Op.	Int.
**GR (IU/g)**	27.56 ± 1.39	15.41 ± 1.95	14.19 ± 2.35	16.45 ± 2.22	66.19 ± 17.50	30.30 ± 7.77	30.53 ± 5.01	14.09 ± 2.29	**<0.001**	**<0.001**	**<0.001**
**CAT (IU/g)**	59.91 ± 3.55	104.42 ± 12.84	84.68 ± 42.72	92.33 ± 9.48	306.63 ± 62.55	93.14 ± 7.01	78.80 ± 14.33	100.82 ± 27.05	**<0.001**	**<0.001**	**<0.001**
**GPx (IU/g)**	15.81 ± 1.23	19.42 ± 1.70	18.76 ± 5.28	18.28 ± 1.82	14.58 ± 4.72	14.76 ± 2.44	15.49 ± 2.44	16.35 ± 1.38	**0.253**	**<0.01**	0.506
**GST (IU/g)**	3.44 ± 0.61	3.74 ± 0.30	2.89 ± 1.08	3.50 ± 0.57	2.66 ± 0.73	3.58 ± 0.82	3.34 ± 0.25	3.43 ± 0.48	0.121	0.632	0.074
**Total SOD (NU/mg)**	79.75 ± 4.71	129.37 ± 27.69	105.22 ± 23.89	93.70 ± 9.57	146.11 ± 6.99	118.33 ± 17.91	77.54 ± 11.64	123.13 ± 26.37	**<0.001**	**<0.01**	**<0.001**
**MnSOD (NU/mg)**	67.40 ± 4.63	89.05 ± 2.89	82.62 ± 20.55	80.50 ± 7.10	45.38 ± 22.15	81.13 ± 16.28	66.17 ± 8.26	69.25 ± 23.10	**<0.001**	**<0.01**	0.700
**CuZnSOD (NU/mg)**	11.96 (11.54–12.74)	42.38 (27.63–49.71)	19.98 (14.80–30.62)	16.47 (12.94–18.51)	16.85 (14.83–18.81)	26.67 (15.80–33.67)	10.34 (8.75–11.82)	59.60 (43.52–93.75)	**<0.001**	0.333	**<0.001**
**MDA (μmol/g)**	3.48 ± 1.33	2.47 ± 0.24	6.10 ± 2.58	2.94 ± 0.79	8.66 ± 1.65	10.53 ± 1.91	2.08 ± 0.78	2.07 ± 0.58	**<0.001**	**<0.001**	**<0.001**

Abbreviations: Group: HF/HF, CD/HF, HF/CD and CD/CD—dietary patterns applied in groups of rats for 8 weeks before and 8 weeks after surgery: HF = high-fat diet; CD = control diet; Op. = type of surgery used in the experiment: DJOS = duodenal-jejunal omega switch, SHAM = control; Int.—interaction within the dietary patterns and type of the surgery. Statistically significant values are bolded.

**Table 2:** Multiple comparisons in contrast analysis. Post hoc analysis of glutathione reductase (GR), catalase (CAT), glutathione peroxidase (GPx), glutathione S-transferase (GST), total superoxide dismutase (SOD), Mn-dependent superoxide dismutase (MnSOD), copper–zinc superoxide dismutase (CuZnSOD) activity and malondialdehyde (MDA) concentration in erythrocytes and heart muscle of rats from study groups subjected to high-fat (HF) and/or to (CD) control diets and subjected to DJOS (duodenal-jejunal omega switch) or SHAM (control) surgery. Statistical significance was set at *p* < 0.05. Statistically significant values are bolded.

Oxidative Stress Markers Post hoc	DJOS vs. SHAM				DJOS						SHAM					
	1: HF/HF	2: HF/CD	3: CD/HF	4: CD/CD	1 vs. 2	1 vs. 3	1 vs. 4	2 vs. 3	2 vs. 4	3 vs. 4	1 vs. 2	1 vs. 3	1 vs. 4	2 vs. 3	2 vs. 4	3 vs. 4
**Erythrocytes**																
**GR (IU/g)**	0.189	**<0.001**	**<0.001**	0.557	**<0.001**	**<0.05**	0.207	**<0.001**	**<0.001**	0.226	0.114	**<0.001**	0.594	**<0.001**	**<0.05**	**<0.001**
**CAT (IU/g)**	**<0.001**	**<0.05**	**<0.001**	**<0.05**	**<0.001**	**<0.001**	**<0.001**	0.056	0.375	0.259	**<0.01**	**<0.001**	**<0.01**	**<0.001**	0.490	**<0.001**
**GPx (IU/g)**	**<0.001**	**<0.01**	0.939	**<0.05**	0.324	**<0.001**	**<0.001**	**<0.01**	**<0.001**	0.180	0.771	0.542	0.094	0.758	0.178	0.296
**GST (IU/g)**	**<0.001**	0.303	**<0.001**	0.178	**<0.001**	**<0.001**	**<0.001**	0.382	**<0.001**	**<0.001**	0.058	**<0.001**	0.127	**<0.001**	**<0.01**	**<0.001**
**Total SOD (NU/mg)**	0.876	0.763	0.857	0.542	**<0.01**	0.181	**<0.001**	0.115	0.293	**<0.01**	**<0.01**	0.156	**<0.01**	0.091	0.898	0.070
**MDA (μmol/g)**	**<0.001**	0.142	0.774	**<0.001**	**<0.001**	**<0.01**	**<0.001**	0.205	**<0.001**	**<0.001**	0.154	0.356	**<0.05**	0.621	**<0.01**	**<0.01**
**Heart Muscle**																
**GR (IU/g)**	**<0.001**	**<0.001**	**<0.001**	0.572	**<0.01**	**<0.01**	**<0.05**	0.771	0.803	0.589	**<0.001**	**<0.001**	**<0.001**	0.956	**<0.001**	**<0.001**
**CAT (IU/g)**	**<0.001**	0.529	0.742	0.635	**<0.05**	0.171	0.075	0.273	0.500	0.669	**<0.001**	**<0.001**	**<0.001**	0.425	0.668	0.222
**GPx (IU/g)**	0.461	**<0.01**	0.064	0.270	**<0.05**	0.095	0.160	0.705	0.513	0.782	0.911	0.586	0.291	0.676	0.361	0.618
**Total SOD (NU/mg)**	**<0.001**	0.300	**<0.05**	**<0.01**	**<0.001**	**<0.05**	0.192	**<0.05**	**<0.01**	0.280	**<0.05**	**<0.001**	**<0.05**	**<0.001**	0.650	**<0.001**
**MnSOD (NU/mg)**	**<0.05**	0.380	0.113	0.215	<0.05	0.096	0.150	0.475	0.343	0.813	**<0.001**	**<0.05**	**<0.01**	0.154	0.190	0.904
**CuZnSOD (NU/mg)**	0.222	**<0.05**	**<0.01**	**<0.001**	**<0.001**	**<0.05**	0.277	**<0.05**	**<0.001**	**0.188**	0.116	**<0.05**	**<0.001**	**<0.001**	**<0.001**	<0.001
**MDA (μmol/g)**	**<0.001**	**<0.001**	**<0.001**	0.303	0.230	**<0.01**	0.514	**<0.001**	0.578	**<0.001**	**<0.05**	**<0.001**	**<0.001**	**<0.001**	**<0.001**	0.990

Column 1: Inter-group comparisons between study groups pre-HF diet vs. post-HF diet, pre-CD vs. post-HF diet, per-HF vs. post-CD and pre-CD vs. post-CD; groups DJOS vs. SHAM. Column 2: Intra-group comparisons between pre-HF diet vs. post-HF diet, pre-CD vs. post-HF diet, pre-HF vs. post-CD and pre-CD vs. post-CD groups after DJOS surgery. Column 3: Intra-group comparisons between pre-HF diet vs. post-HF diet, pre-CD vs. post-HF diet, pre-HF vs. post-CD and pre-CD vs. post-CD groups after SHAM surgery.

## Data Availability

The original data are available after contact with the corresponding author.
